# Phenotypic and Genetic Correlations Between the Lobar Segments of the Inferior Fronto-occipital Fasciculus and Attention

**DOI:** 10.1038/srep33015

**Published:** 2016-09-06

**Authors:** Yuan Leng, Yonggang Shi, Qiaowen Yu, John Darrell Van Horn, Haiyan Tang, Junning Li, Wenjian Xu, Xinting Ge, Yuchun Tang, Yan Han, Dong Zhang, Min Xiao, Huaqiang Zhang, Zengchang Pang, Arthur W. Toga, Shuwei Liu

**Affiliations:** 1 Research Center for Sectional Imaging Anatomy, Shandong Provincial Key Laboratory of Mental Disorders, Shandong University School of Medicine, 44 Wen-hua Xi Road, 250012 Jinan, Shandong, China; 2 Laboratory of Neuro Imaging, USC Mark and Mary Stevens Neuroimaging and Informatics Institute, Keck School of Medicine of University of Southern California, Los Angeles, CA 90033, USA; 3 Department of Radiology, Affiliated Hospital of Medical College, Qingdao University, 266003 Qingdao, Shandong, China; 4 Department of Epidemiology, Qingdao Municipal Center for Disease Control and Prevention, 266033 Qingdao, Shandong, China

## Abstract

Attention deficits may present dysfunctions in any one or two components of attention (alerting, orienting, and executive control (EC)). However, these various forms of attention deficits generally have abnormal microstructure integrity of inferior fronto-occipital fasciculus (IFOF). In this work, we aim to deeply explore: (1) associations between microstructure integrities of IFOF (including frontal, parietal, temporal, occipital, and insular segments) and attention by means of structural equation models and multiple regression analyses; (2) genetic/environmental effects on IFOF, attention, and their correlations using bivariate genetic analysis. EC function was attributed to the fractional anisotropy (FA) of left (correlation was driven by genetic and environmental factors) and right IFOF (correlation was driven by environmental factors), especially to left frontal part and right occipital part (correlation was driven by genetic factors). Alerting was associated with FA in parietal and insular parts of left IFOF. No significant correlation was found between orienting and IFOF. This study revealed the advantages of lobar-segmental analysis in structure-function correlation study and provided the anatomical basis for kinds of attention deficits. The common genetic/environmental factors implicated in the certain correlations suggested the common physiological mechanisms for two traits, which should promote the discovery of single-nucleotide polymorphisms affecting IFOF and attention.

The inferior fronto-occipital fasciculus (IFOF) connects various parts of the occipital cortex, temporo-basal area, and the superior parietal lobule to the frontal lobe through the external/extreme capsule complex^1^,^2^. It provides anatomical connectivities for spatial attention^3^,^4^,^5^, especially in the ventral attention system^6^.

Although direct fronto-parietal connection is crucial for attention^7^,^8^, there remains an incomplete understanding about its importance for the three subnetworks of attention: alerting, orienting, and executive control (EC), respectively. The study of chronic visual neglect suggested that damage to fronto-parietal connections in the right hemisphere was important for orienting of spatial attention^9^. However, Vallar *et al.* proposed that the parieto-frontal connections of IFOF were not involved in the orientation of attention^10^.

According to previous disease studies, abnormal microstructural integrity of IFOF had been linked with attention deficit hyperactivity disorder (ADHD)^11^, autism spectrum disorders (ASD)^12^, and schizophrenia^13^. While in function, children with ADHD had executive dysfunction^14^ and/or alerting deficits^15^; young children with ASD showed intact alerting attention, but were less-efficient in orienting and EC^16^; patients with schizophrenia had specific deficit in executive control of attention^17^. The varieties led us to explore whether these attentional deficits resulted from the abnormal white matter integrity of IFOF.

Identification of genetic factors affecting white matter integrity and cognitive functions is vital important in neuroscience. However, little is known about the heritability of IFOF or attention. Brouwer *et al.* suggested that the heritability of IFOF was 7–35% in early puberty (9 and 12 year olds) and the genetic factors on the variation of FA increased with age^18^. A study comparing adolescents (12 and 16 year olds) to adults (aged 23.7 ± 2.1) showed that the FA of right inferior longitudinal fasciculus (ILF)/IFOF was more heritable in the younger group (70–80%) than in adults (30–40%)^19^. However, that study failed to distinguish IFOF from ILF and to elucidate the heritability of IFOF in late adolescents (16 to 20 year olds). Likewise, the genetic effects on the three subnetworks of attention were also under debate^20^,^21^.

Quantitative tractography has the advantages of mapping the direction of white matter fibers and provides a unique opportunity to study white matter architecture *in vivo*^22^. Using diffusion tractography, our previous research suggested that the white matter asymmetry of IFOF in frontal lobe was correlated with the EC of attention^23^, which might indicate that the parts of IFOF in different lobes were related to specific subnetwork of attention. Hence, for a more detailed analysis, we divided the IFOF into five segments based on the brain regions: frontal, parietal, occipital, temporal, and insular.

Our research aims to calculate the white matter integrities FA and mean diffusivity (MD) of the IFOF, as well as the five specific segments, using the quantitative tractography method, and to correlate these attributes with the three subnetworks of attention. Furthermore, based on the advantages of twin study design, we have a chance to clarify the genetic and environmental impact on the correlations between the two traits. With the evidence that EC alternation is an important neuropsychological endophenotype in schizophrenia^24^ and ADHD^25^, and the fact that IFOF plays a crucial role in these two diseases, we hypothesize that the white matter integrities of IFOF are correlated with EC component.

## Results

### Behavioral results

The accuracy of ANT performance was 80.1 ~ 99.5% (averaged = 96.6%) and no one was excluded from the study, indicating that the participants understood the behavioral task and could make a reliable determination on the direction of the central arrow.

The correlations between the ratio scores of alerting, orienting and EC are shown in Table 1. Only the significant correlation between alerting and orienting was found. There was no gender difference in ratio scores.

### The IFOF and its segments

**Figure 1:**
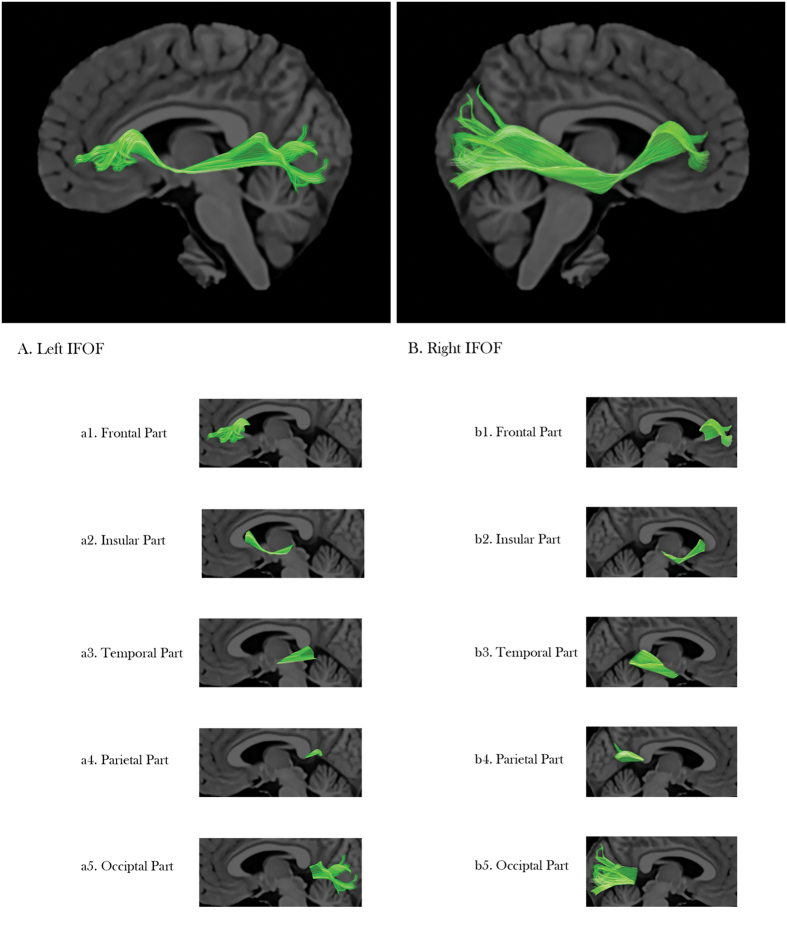
The whole IFOF and its lobar segments. (**A**) is the left IFOF, a1 to a5 is the frontal part, insular part, temporal part, parietal part, and occipital part, respectively. (**B**) is the right IFOF; b1 to b5 is the frontal part, insular part, temporal part, parietal part, and occipital part, respectively.

The main courses of the left and right IFOF, as well as their lobar segments, were shown in Fig. 1. Mean FA and MD of the left IFOF was 0.4350 ± 0.0314 and 0.8349 ± 0.0329 (*10^−3^), respectively. Using quantitative tractography, high intra-rater reliability of 0.96 was obtained for the left IFOF. Mean FA and MD of the right IFOF was 0.4497 ± 0.0185 and 0.8201 ± 0.0293 (*10^−3^), respectively. The intra-rater reliability for the right IFOF was 0.97 in our study.

### Statistical results

**Figure 2:**
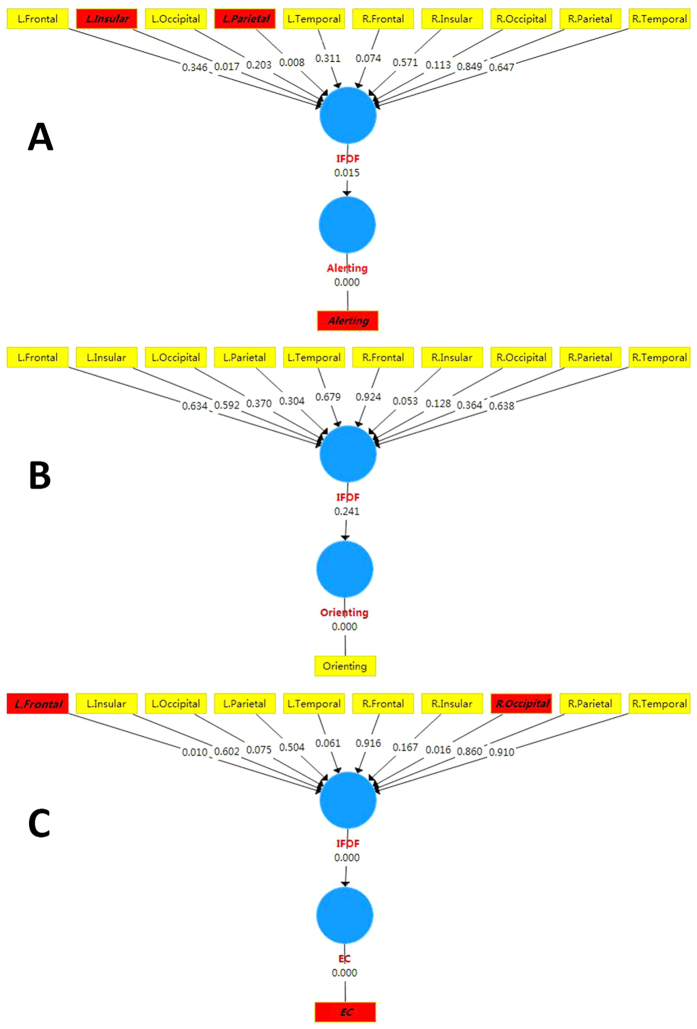
The p values of path coefficients of the structural models. (**A**–**C**) represented the associations of FA in segmented IFOF to alerting, orienting, and EC, respectively. The parameters with red background indicated the significant correlations.

According to the p values of the path coefficients in structural models, we found the significant effects of FA in the left insular and parietal parts on alerting (Fig. 2A) and the effects of the right occipital part and left frontal part on EC (Fig. 2C). No significant correlations were found between the FA in segments of IFOF and orienting (Fig. 2B). There were no associations of MD in segments of IFOF to attention.

**Figure 3:**
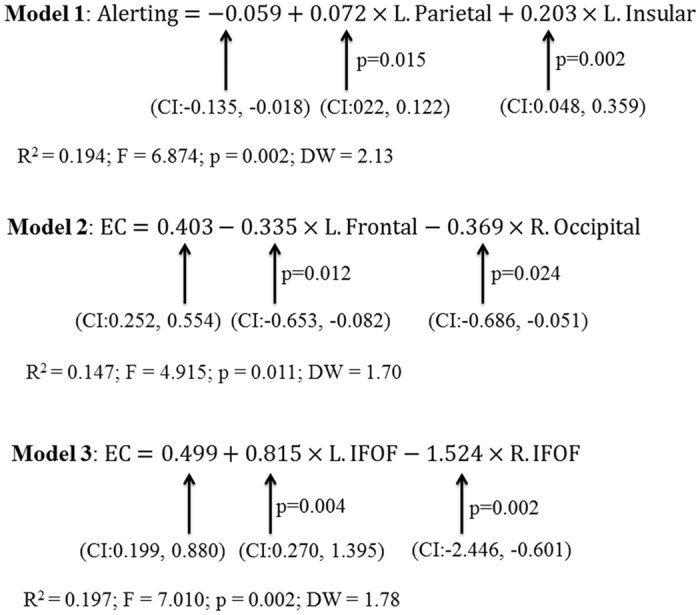
The multiple regression analyses for attention and FA of IFOF, as well as its segments. Model 1 to Model 3 represented the multiple regression equations of alerting and the segments of IFOF, EC and segments of IFOF, and EC and the whole IFOF, respectively. Abbreviations: L, Left; R, Right; IFOF, Inferior Fronto-Occipital Fasciculus; EC, Executive Control; CI, 95% Confidence Interval; DW, Durbin-Watson.

The correlations between different parts of the tract were low and no significant multicollinearity was found. The results of multiple regression models for FA of IFOF were shown in Fig. 3. In the segmental analysis, the insular and parietal segments of left IFOF were positively correlated with alerting ratio scores (Fig. 3, Model 1, p = 0.002); both the right occipital part and left frontal part were negatively correlated with EC ratio scores (Fig. 3, Model 2, p = 0.011); no significant correlation was found between the FA in segmented IFOF and orienting. For the FA of whole IFOF, there were no relationships between IFOF and alerting, as well as orienting; the left and right IFOF was positively and negatively correlated with EC ratio scores, respectively (Fig. 3, Model 3, p = 0.002). The multiple regression analyses were not performed for MD of IFOF because of the lack of significant associations to attention in aforementioned structural model. Hence, the alpha level of the three components of attention was corrected to 0.025 (p < 0.05 divided by two times of repetitions). Using this criterion, the significant correlations remained between IFOF and alerting, as well as EC.

### Heritability results

Heritability results were shown in Table 2. According to the comparison of hierarchical models (see Supplementary Tables S1–S5), the best model was chosen for each parameter. The genetic influence on the FA of IFOF was about 40–54% in late adolescence. The heritability of the FA in left IFOF was higher than that of the right one, which was opposite to the MD. Additive genetic factors contributed to both orienting and EC, while no evidence of heritability of alerting was found in our study.

**Figure 4:**
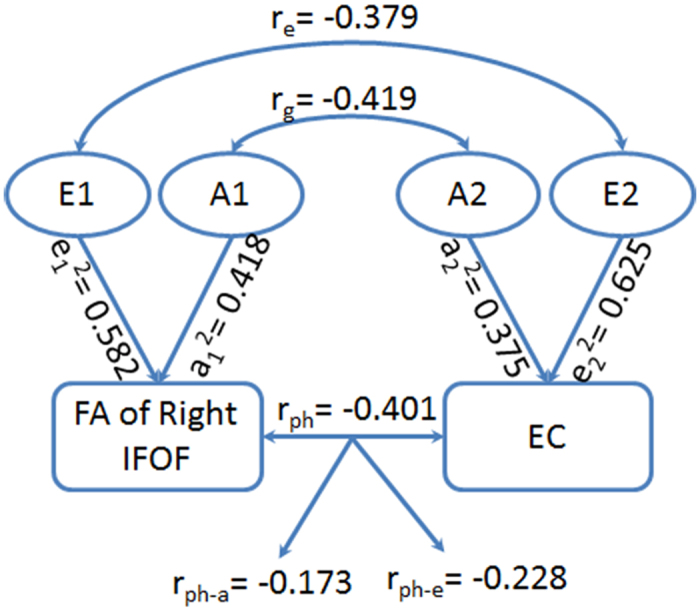
Phenotypic (r_ph_), genetic (r_g_), and environmental (r_e_) correlations between the FA of right IFOF and EC, as well as the additive genetic and specific environmental estimates for the right IFOF (a_1_^2^ and e_1_^2^), EC (a_2_^2^ and e_2_^2^), and their correlation (r_ph-a_ and r_ph-e_).

The results of the bivariate genetic analysis were shown in Table 3 and Fig. 4. EC ratio scores were phenotypically correlated with FA of left IFOF (r_ph_ = 0.208) and the right occipital part (r_ph_ = −0.266); genetic factors were implicated in these correlations (r_g_ = 0.916, r_g_ = −0.570) and the extent of phenotypic correlation due to gene was 0.452 and −0.168, respectively. The phenotypic and environmental correlations were found between EC and the right (r_ph_ = −0.401, r_e_ = −0.379) and left IFOF (r_ph_ = 0.208, r_e_ = −0.555); the extent of phenotypic correlation due to environmental factors was −0.228 and −0.244, respectively. None of the other phenotypic correlations were found to be driven by significant genetic or environmental factors.

## Discussion

The FA value may reflect the fiber density, axonal diameter, and myelination in white matter^26^. Our failure to observe the significant correlation for MD might suggest that FA was a more sensitive biological marker for IFOF. Higher EC ratio scores mean longer reaction time and lower ability in executive control. In this study, the left IFOF was negatively associated with EC function and this relationship was driven by genetic and environmental factors; the right IFOF was positively correlated with EC function and such relationship was only driven by environmental factors; the left frontal part and right occipital part of IFOF were both associated with EC function and the latter relationship was driven by genetic factors. The insular and parietal segments of left IFOF were positively correlated with alerting and no significant genetic or environmental influences were found on these relationships. Compared with the non-significant correlation between alerting and the whole IFOF, the association of segmented IFOF to alerting might indicate the advantages of segmental analysis in structure-function correlation study.

The alerting component during ANT task, which was defined as phasic or exogenous alertness, represented the ability to activate the required cognitive systems to make the person ready to respond to a task^27^. In our study, alerting was positively correlated with FA in the insular and parietal parts of the left IFOF. For the parietal segment, previous fMRI studies focused on noradrenergic modulation have suggested the activity of alerting in the inferior and superior parietal cortex^28^. We first demonstrated that the left IFOF in the insular lobe provided structural connectivity for alerting network. The insula showed connections with the frontal, temporal, parietal, and thalamic regions^29^ that had been demonstrated to modulate the alerting network^30^,^31^,^32^. Notably, Ghaziri *et al.* revealed the clear structural connectivity between the insular cortex and cingulate cortex^33^. The anterior cingulate cortex had been reported to play a critical role in state maintenance^34^, which is important for alerting function. However, our study was limited to depict the IFOF in the insula, more studies will be needed to resolve the functional connections between insular cortex and other regions.

Our findings revealed the importance of left hemisphere in alerting, which is consistent with some prior studies, while opposite to others. By means of DTI and region of interest (ROI) analysis, Niogi *et al.* found the significant structure–function correlations between alerting and the left posterior limb of the internal capsule^27^, which is located medially to the insula. Similarly, previous fMRI study discovered that the alerting effect primarily activated a left-lateralized fronto-parietal network of areas^28^. However, for patients with chronic schizophrenia, the FA of left cingulum bundle correlated with orienting of attention and smaller right cingulum bundle volume correlated with reduced alertness^35^. These inconsistencies might be resulted from methodology, subjects or the overlaps between alerting and orienting.

The orienting system had been reported to be associated with frontal eye fields, superior and inferior parietal lobes, the superior colliculus, and reticular nuclei^31^,^36^,^37^. It must relay and compare spatial information from both visual fields that requires connectivity between hemispheres^27^. Previous lesions and fMRI studies have found the modulatory role of commissural fibers in the function of orienting network^38^,^39^. In our study, we did not find the significant correlation between orienting and the IFOF in different brain regions. This finding is consistent with Vallar *et al.*’s report of a neglect study that proposed the inefficiency of IFOF in the orientation of attention^10^. However, the white matter damages involving IFOF and superficial damage to the inferior parietal cortex were found in two patients with neglect^40^, a deficit that has effects on orienting functions of attention. These different results might be related to the different fiber tracking methods. The anatomical course of IFOF is complex and hard to be isolated from the surrounding fiber tracts, such as ILF, which has been described in neglect patients^41^.

Executive control is involved in the resolution of incongruent stimuli impacting decision planning and making. This network includes the white matter tracts that connect the frontal lobe with other regions^32^,^36^,^42^. Multiple regression analysis in our study revealed the association of EC ratio scores to the FA of left and right IFOF, indicating that higher EC ability increased with higher FA in the right IFOF and the lower FA in the left IFOF. These findings might suggest that EC function was correlated with the rightward asymmetry of IFOF, which is coincided with the theory of rightward asymmetry for sustained attention^43^. It is thought that the inhibitory control process within the right inferior frontal gyrus could enhance the cognitive efficiency by emphasizing the required response and inhibiting the irrelevant stimulus^44^. In addition, a previous model-based fMRI study also revealed higher FA in the right IFOF for good performance in selective response inhibition^45^. Our findings suggested that EC function was associated with the part of IFOF in occipital lobe. In patients with multiple sclerosis, better cognitive performance was correlated with increased functional connectivity between anterior cingulate cortex and occipital lobe^46^. Moreover, the decreased functional connectivity between the anterior cingulate cortex and occipital lobe was associated with the increase in executive reaction time^47^.

Besides, DTI studies have addressed the importance of left frontal white matter tracts in attention. Niogi *et al.* found that the association between FA values within a ROI in anterior corona radiata (ACR) and EC was significant in the left hemisphere and appeared a non-significant trend in the right one^27^. However, they failed to figure out the certain fiber because of the mixture of projection, association, and callosal fibers in ACR area. In patients with mild traumatic brain injury, the FA of left ACR was shown to correlate with conflict scores of ANT^48^. A previous neuropsychological study showed that executive function was negatively correlated with MD of the left IFOF in bipolar disorder^49^. However, there was no association of EC to the MD of left IFOF in our study, indicating less sensitivity of MD than that of FA in structure-function correlation analysis in healthy subjects.

Different from earlier developing structures that are under higher genetic control, tract-level heritability was not modulated by age and the earlier developing white matter tracts did not show a higher degree of genetic contribution^50^, which may be related to the different functions that fibers participate in. Previous researches focused on adults or children cannot be used as a comparator for teenagers. Our result provides important information about the heritability of the IFOF in late adolescence (40–54%). Previous studies suggested the heritability of IFOF is at 7–35% in early puberty^18^, 70–80% in young adolescents, and 30–40% in adults^19^. Combining with our study, we speculate that the heritability of IFOF has begun to peak off since early adolescence. As with the heritability of white matter tract, the genetic effects on cognitive function differ from age to age. Previous studies with a wide age period showed a high heritability in the conflict network, low heritability in alerting, and no heritability in orienting^21^. A twin study with the mean age of 50 suggested that the heritability of orientation was about 0.38^20^. In our research, alerting showed non-significant heritability in adolescents. In the future, we will enrich our sample size to clearly clarify the heritability of IFOF during this period.

In addition, determining the extent of genetic or environmental influence on structure-function correlations enhances our knowledge about brain morphology eventually contributing to human behavior. Previous studies have demonstrated the genetic correlations between microstructural properties of white matter tracts and intelligence^51^. Our results suggested that common genes were implicated in the relationship between EC and the left IFOF, as well as the right occipital part. Both EC and IFOF were affected by kinds of gene variations. Hence, we speculated that not a specific gene but the results of some genes interaction modulated the correlation between white matter tract and cognitive function. However, this study was limited to evidence the extent of genetic overlaps between IFOF and attention. GWAS with much more subjects will further explain the certain genes that related to both IFOF and three subnetworks of attention in the future.

Although these findings are robust, some limitations still need to be addressed. First, estimates of the eigenvector directions, and hence the local tract directions, are sensitive to thermal noise, physiologic fluctuations, and image artifacts. Algorithms based on the major eigenvector are unable to resolve regions of crossing white matter pathways^26^. Other diffusion imaging methods, such as High Angular Diffusion Imaging (HARDI)^52^, may be used to get over the junctions problem more accurately. Second, DTI tractography is based on the course of IFOF and pictures two ROIs on its course. After that, we need some NOT applications, which mainly based on the anatomical knowledge and prior experience. Although we have verified the reliability and reproducibility of our results, the artificial factors may still take some disturbance to our results. Third, given the small sample size in our study, we cannot be certain that the genetic correlations between IFOF and attention would be similar in a greater sample size. However, the heritability result was coincident with the law of changes of heritability. It might be sufficient for such exploratory work to introduce the trend of genetic effects on brain structure, function, and their correlation during late adolescence. It remains an issue for future research to determine if the genetic relationship observed in this small sample may be unique to this age period.

## Conclusions

White matter tract segmentation provided a new sight into the brain structure-function correlations. In this study, we were able to identify the role of lobar-segmented IFOF in attention. Our segmental analysis suggested the phenotypic contribution of IFOF in the left frontal lobe and right occipital lobe to EC function and that of IFOF in the left insular and parietal lobes to alerting, which provided anatomical basis for alerting and EC deficits. Moreover, due to the small sample size, genetic factors were only implicated in the right occipital part and EC association. Future studies with much more subjects and multi-model methods should be applied to investigate the underlying explanation for the associations between lobar segments of IFOF and three subnetworks of attention.

## Materials and Methods

### Subjects

60 healthy subjects (24 males, 36 females; 14 pairs MZ, 16 pairs DZ) with 15–20 years of age (mean age: 16.9 ± 1.53 years old) were recruited for the study. All were Chinese native speakers with normal or corrected-to-normal vision. Inclusion criteria were: (1) right-handed measured with Edinburgh Inventory^53^; (2) no history of neuropsychiatric disease and no abnormities in the conventional brain MR images. This study was conducted on the basis of approval from the Human Research Ethics Committees of the Shandong University School of Medicine. All procedures were carried out in accordance with the approved guidelines. All participants as well as their parents provided written informed consent.

### Behavioral task

The ANT task was adopted as a cognitive task in our study^42^. It has the advantage of evaluating the efficiency of alerting, orienting, and EC in a single integrated task. The test started with a cue, which was shown as an asterisk. The cue was presented in three conditions: no cue, center cue, and spatial cue. Two hundred milliseconds later, a left or right arrow (the target) was shown at the center of the screen and flanked by two arrows on either side in the same direction (congruent condition), or the opposite direction (incongruent condition). Subjects were instructed to press a button to make a decision about the direction of the central target arrow as quickly and accurately as possible. Both the target and flankers disappeared once the participant responded or 2000 ms elapsed. Each subject performed a total of six trial blocks. Each block consisted of thirty-six trials and lasted for 5 minutes and 42 seconds. All subjects were trained by a specialist just before the formal performance. E-Prime (Psychology Software Tools, Pittsburgh, PA) was used to carry out the stimulus presentation and behavioral response collection.

### Behavioral data analysis

The reaction time (RT) and total accuracy of each subject were calculated. The participant with low accuracy (<80%) should be excluded from our study. Trials with incorrect responses or RTs shorter than 200 ms or longer than 1,500 ms were also excluded to avoid the influence of the abnormal values. Responses following erroneous ones were removed to avoid post-error slowing effect. In this study, we used ratio scores of alerting, orienting, and EC to define the efficiency of three components of attention. The formulas were as follows:


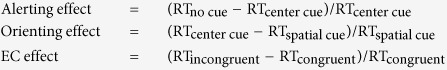


### MRI data acquisition

MR imaging was performed on a 3.0 T GE Signa scanner (General Electric Medical Systems, Milwaukee, WI). The spin-echo, single shot echo planar imaging sequence was used to acquire the diffusion MR image with the following parameters: TR, 14,000 ms; TE, 75.1 ms; field of view (FOV), 250 × 250 mm^2^; matrix, 96 × 96; slice thickness, 2.6 mm with no gap; slice number, 56. 30 non-colinear diffusion gradients directions (b = 1,000 s/mm^2^) and 3 non-diffusion-weighted images (b = 0 s/mm^2^) were included in the DTI scans. Array spatial sensitivity encoding technique (ASSET) was used with an acceleration factor of 2. The sequence was repeated twice to increase signal-to-noise.

After the DTI scans, the structural images were collected using a three-dimensional spoiled gradient-echo (SPGR) pulse sequence: TR, 6.5 ms; TE, 2.0 ms; FOV, 256 × 256 mm^2^; matrix, 256 × 256; flip angle, 15°; slice thickness, 1.0 mm with no gap; slice number, 174. Both the diffusion MR and structural images collections were the same to our prior study^23^.

### DTI data analysis

DTI data processing was performed using the Laboratory of Neuro Imaging (LONI) Pipeline Workflow Environment (http://pipeline.loni.usc.edu; version 6.0). First the DTI data were preprocessed using the FSL toolbox^54^. The diffusion data were corrected for eddy currents and head motion, and the two acquisitions were averaged. Fiber reconstruction was performed using the Diffusion Toolkit^55^ based on a streamline algorithm. It trims any fibers that bend greater than 30 degrees to reduce the mis-identification of fibers. The track visualization and track extraction was performed using TrackVis (http://www.trackvis.org). To ensure reliability, we adopted a multi-ROIs approach to extract the IFOF manually. The first ROI delineated the occipital lobe on a coronal slice, which was identified at the middle point between the posterior edge of the parieto-occipital sulcus and the posterior edge of the cingulum. The second ROI was also selected on a coronal slice, which located at the anterior edge of the genu of corpus callosum and delineated the entire hemisphere^56^. Once the “AND” operation was employed, the whole fasciculus was obtained. If a tract was clearly anatomically incorrect, the NOT function was used to remove the fiber from the bundle. Inter- and intra-class reliability were calculated by intra-class correlation coefficients of integrities in the left and right IFOF.

### Lobar segments of the IFOF

To clarify the function of the IFOF in different brain regions, we divided it into five segments according to the brain lobes: frontal, parietal, temporal, occipital, and insular. To do this, the MNI structural atlas was registered to the MR images by Advanced Normalization Tools (ANTs)^57^. Nearest neighbor interpolation was then applied to obtain the lobar label at each point of the fiber bundle. After that the lobar segments of the IFOF were obtained by clustering points with the same lobar labels.

### Statistical analysis

Advanced multivariate statistical software of Structural Equation Modelling (SmartPLS v3.0) (http://www.smartpls.de) was preliminary used to assess the effect of FA in manifest variables (segments of IFOF) on alerting, orienting, and EC, respectively. Structural model assesses the relationships through evaluating the path coefficients (β value) of the model. The path coefficient has to be tested for its significant level by *t*-value test (two-tailed), which is achieved by bootstrapping technique. The same procedure was repeated for MD of segmented IFOF.

To further explain the extent of the possible causal linkage among the statistical variables, multiple regression analyses for IFOF and attention were performed in the Statistical Package for Social Sciences (SPSS), version 20.0 (SPSS Inc., Chicago, IL, USA). As a matter of fact, one variable in a multiple regression model can be linearly predicted from the others with some certain degree of accuracy and the coefficient estimates of the multiple regressions may be changeful due to small changes in the model or the data, multicollinearity was used to test whether two or more variables were highly correlated. Subsequently, ten blocks of independent variables were applied in a step-wise fashion. Likewise, multiple regression analyses were performed to test the specificity of the whole IFOF to the three attention subnetworks. A Bonferroni correction was applies for multiple comparisons and the alpha level was set to 0.05 divided by the times of repetitions for each attention component.

### Heritability analysis

Heritability calculation was performed using the OpenMx package (http://openmx.psyc.virginia.edu) in the R statistical computing environment (http://www.r-project.org). Phenotypic variance in the twin genetic model was estimated by the contribution of three factors: additive genetic factors (A), common environment factors (C) and specific environment factors (E), which could be called ACE model. A full ACE model was compared with an AE-model, a CE-model, or an E-model. The goodness of fit of different models was evaluated by Chi-square differences and the Akaike’s Information Criterion (AIC)^58^.

The bivariate genetic analysis yielded an estimate of the phenotypic correlations (r_ph_) between attention and the IFOF, which can result from genes or environmental factors. The extent of the overlap is reflected by the genetic and environmental correlation r_g_ and r_e_, respectively. In addition, combining the genetic and environmental correlations with the heritability of each trait, we also established the genetic (r_ph-a_) and environmental (r_ph-e_) contributions to the phenotypic correlation between the two traits^59^.

## Additional Information

**How to cite this article**: Leng, Y. *et al.* Phenotypic and Genetic Correlations Between the Lobar Segments of the Inferior Fronto-occipital Fasciculus and Attention. *Sci. Rep.* **6**, 33015; doi: 10.1038/srep33015 (2016).

## Supplementary Material

Supplementary Information

## Figures and Tables

**Table 1:** The ratio scores (Mean ± SD) of three components of attention and their correlation coefficients.

	Sample size	Alerting	Orienting	EC
MZ	**28**	0.064 ± 0.040	0.092 ± 0.061	0.164 ± 0.075
DZ	**32**	0.056 ± 0.038	0.090 ± 0.47	0.172 ± 0.056
*t*(P)		0.723 (0.472)	0.119 (0.905)	0.459 (0.647)
Alerting	**60**	**1**		
Orienting	**60**	−0.371 (0.003)*	**1**	
EC	**60**	**0.041 (0.751)**	**0.023 (0.860)**	**1**

*t*, the *t* value of independent samples *t*-test (two-tailed). The numbers in parentheses represent P values of statistical analyses. * = significant correlation, p < 0.05. EC, executive control.

**Table 2:** Heritability of white matter integrities (FA and MD) in the IFOF and three subnetworks of attention.

	Left		Right	
	a^2^ (95% CI)	e^2^ (95% CI)	a^2^ (95% CI)	e^2^ (95% CI)
*FA*				
IFOF	0.539 (0.097, 0.796)	0.461 (0.203, 0.903)	0.418 (0.089, 0.715)	0.582 (0.285, 0.911)
Frontal	0 (0, 0.412)	1 (0.507, 1)	0.247 (0.007, 0.559)	0.753 (0.411, 0.993)
Insular	0.269 (0, 0.560)	0.731 (0.439, 1)	0.239 (0.019, 0.592)	0.761 (0.403, 0.980)
Temporal	0.276 (0.019, 0.577)	0.723 (0.422, 0.981)	0.294 (0.025, 0.606)	0.706 (0.393, 0.974)
Parietal	0 (0, 0.247)	1 (0.695,1)	0.157 (0.004, 0.467)	0.843 (0.533, 0.996)
Occipital	0.485 (0.012, 0.757)	0.515 (0.243, 0.953)	0.231 (0.098, 0.551)	0.769 (0.448, 0.902)
*MD*				
IFOF	0.332 (0.002, 0.698)	0.668 (0.301, 1)	0.424 (0.013, 0.720)	0.576 (0.279, 0.983)
Frontal	0.491 (0.012, 0.732)	0.509 (0.268, 0.891)	0.479 (0.049, 0.738)	0.521 (0.262, 0.959)
Insular	0.059 (0, 0.886)	0.941 (0.113, 1)	0 (0,0.432)	1 (0.567, 1)
Temporal	0.620 (0.018, 0.809)	0.380 (0.191, 0.716)	0.365 (0.027, 0.667)	0.635 (0.333, 1)
Parietal	0.250 (0, 0.649)	0.750 (0.350, 1)	0.229 (0.014, 0.558)	0.771 (0.441, 1)
Occipital	0.367 (0, 0.695)	0.633 (0.304, 1)	0 (0, 0.292)	1 (0.669, 1)
	a^2^ (95% CI)	e^2^ (95% CI)		
Alerting	0 (0, 0.418)	1 (0.581, 1)		
Orienting	0.464 (0.122, 0.736)	0.536 (0.057, 0.942)		
EC	0.375 (0.084, 0.677)	0.625 (0.323, 0.915)		

The best model was chosen for each parameter (based on the AIC and Chi-square differences). Abbreviations: IFOF, inferior fronto-occipital fasciculus; FA, fractional anisotropy; MD, mean diffusivity.

**Table 3:** Significant phenotypic correlations between the IFOF (including segments) and three subnetworks of attention and the decomposed sources of these correlations (non-significant phenotypic correlations are excluded from the table).

	r_ph_ (95% CI)	r_g_ (95% CI)	r_e_ (95% CI)	r_ph-a_	r_ph-e_
*EC*					
FA of right IFOF	−0.401 (−0.616, −0.080)	−0.419 (−1, 1)	−0.379 (−0.716, −0.172)	−0.173	−0.228
FA of left IFOF	0.208 (0.023, 0.379)	0.916 (0.502, 1)	−0.555 (−0.806, −0.087)	0.452	−0.244
FA of right occipital part	−0.266 (−0.481, −0.048)	−0.570 (−1, −0.287)	−0.140 (−0.674, 0.226)	−0.168	−0.098
FA of left frontal part	−0.257 (−0.459, −0.072)	0.013 (−1,1)	−0.325 (−0.625, 0.324)	0	−0.257
*Alerting*					
FA of left parietal part	0.312 (0.066, 0.511)	0.99 (−1, 1)	0.310 (−0.075, 0.662)	0	0.310
FA of left insular part	0.280 (0.058, 0.483)	−0.99 (−1,1)	0.008 (−0.405, 0.455)	0	0.007

r_ph_, r_g_, and r_e_ indicate the phenotypic, genetic, and environmental correlations, respectively. r_ph-a_ and r_ph-e_ indicate the phenotypic correlations due to genetic and environmental influence, respectively. The 95% CIs including 0 indicate statistical nonsignificant. Abbreviations: EC, executive control; IFOF, inferior fronto-occipital fasciculus; FA, fractional anisotropy.
