# Supplementary material for: Phenotypic and Genetic Correlations Between the Lobar Segments of the Inferior Fronto-occipital Fasciculus and Attention
Source: Sci Rep. 2016 Sep 6;6:33015. doi: 10.1038/srep33015 (PMC5011720; doi:10.1038/srep33015)
Supplement: Supplementary Information [file srep33015-s1.pdf]

Phenotypic and Genetic Correlations Between the Lobar Segments of the Inferior Fronto-occipital Fasciculus and Attention

Yuan Leng [a,b], Yonggang Shi [b], Qiaowen Yu [a], John Darrell Van Horn [b], Haiyan Tang [a], Junning Li [b], Wenjian Xu [c], Xinting Ge [a], Yuchun Tang [a] , Yan Han [c], Dong Zhang [d], Min Xiao [a], Huaqiang Zhang [d], Zengchang Pang [d], Arthur W. Toga [b,*], Shuwei Liu [a,**]

Table S1: Heritability of FA in the left IFOF and its lobar segments.

	Model	ep	-2LL	AIC	p	a^2	c^2	e^2	Best model
**FA of left IFOF**	ACE	4	-251.54	-363.54	-	0.539	0	0.461	AE
	CE	3	-248.83	-362.83	0.10	-	0.301	0.699	
	AE	3	-251.54	-365.54	0.99	0.539	-	0.461	
**FA of left frontal part**	ACE	4	-186.33	-298.33	-	0	0	1	E
	CE	3	-186.33	-300.33	0.98	-	0	1	
	AE	3	-186.33	-300.33	1.00	0	-	1	
	E	2	-186.33	-302.33	1.00	0	0	1	
**FA of left insular part**	ACE	4	-170.36	-282.35	-	0.269	0	0.731	AE
	CE	3	-170.07	-284.07	0.60	-	0.387	0.613	
	AE	3	-170.36	-284.35	1.00	0.269	-	0.731	
**FA of left temporal part**	ACE	4	-159.49	-271.48	-	0.276	0	0.724	AE
	CE	3	-159.40	-273.40	0.77	-	0.268	0.732	
	AE	3	-159.49	-273.48	1.00	0.276	-	0.724	
**FA of left parietal part**	ACE	4	-32.03	-144.02	-	0	0	1	E
	CE	3	-32.03	-146.02	1.00	-	0	1	
	AE	3	-32.03	-146.02	1.00	0	-	1	
	E	2	-32.03	-148.02	1.00	-	-	1	
**FA of left occipital**	ACE	4	-206.95	-318.95	-	0.485	0	0.515	AE

**part**	CE	3	-205.64	-319.64	0.25	-	0.326	0.674
	AE	3	-206.95	-320.95	1.00	0.485	-	0.515

Table S2: Heritability of FA in the right IFOF and its lobar segments.

	Model	ep	-2LL	AIC	p	a^2	c^2	e^2	Best model
**FA of right IFOF**	ACE	4	-312.54	-424.54	-	0.315	0.088	0.597	AE
	CE	3	-312.28	-426.28	0.61	-	0.428	0.572	
	AE	3	-312.51	-426.51	0.86	0.418	-	0.582	
**FA of right frontal part**	ACE	4	-228.25	-340.25	-	0.247	0	0.753	AE
	CE	3	-227.48	-341.48	0.38	-	0.276	0.724	
	AE	3	-228.25	-342.25	1.00	0.247	-	0.753	
**FA of right insular part**	ACE	4	-211.90	-323.90	-	0.239	0	0.761	AE
	CE	3	-211.77	-325.77	0.85	-	0.195	0.805	
	AE	3	-211.90	-325.90	1.00	0.239	-	0.761	
**FA of right temporal part**	ACE	4	-207.05	-319.05	-	0.294	0	0.706	AE
	CE	3	-206.14	-320.14	0.34	-	0.304	0.696	
	AE	3	-207.05	-321.05	1.00	0.294	-	0.706	
**FA of right parietal part**	ACE	4	-37.92	-149.92	-	0.157	0	0.843	AE
	CE	3	-37.41	-151.41	0.48	-	0.204	0.796	
	AE	3	-37.92	-151.92	1.00	0.157	-	0.843	
**FA of right occipital part**	ACE	4	-197.04	-309.04	-	0.231	0	0.769	AE
	CE	3	-196.31	-310.31	0.40	-	0.262	0.738	
	AE	3	-197.04	-311.04	1.00	0.231	-	0.769	

Table S3: Heritability of MD in the left IFOF and its lobar segments.

	Model	ep	-2LL	AIC	p	a^2	c^2	e^2	Best model
**MD of left IFOF**	ACE	4	-238.00	-350.00	-	0.332	0	0.668	AE
	CE	3	-237.74	-351.74	0.46	-	0.245	0.755	
	AE	3	-238.00	-352.00	1.00	0.332	-	0.668	
**MD of left frontal part**	ACE	4	-171.28	-283.28	-	0.491	0	0.509	AE
	CE	3	-169.79	-283.79	0.22	-	0.469	0.531	
	AE	3	-171.28	-285.28	1.00	0.491	-	0.509	
**MD of left insular part**	ACE	4	-206.13	-318.13	-	0.059	0	0.941	AE
	CE	3	-206.11	-320.11	0.89	-	0	1.000	
	AE	3	-206.13	-320.13	1.00	0.059	-	0.941	
**MD of left temporal part**	ACE	4	-197.77	-309.76	-	0.620	0	0.380	AE
	CE	3	-196.98	-310.98	0.68	-	0.552	0.448	
	AE	3	-197.77	-311.76	1.00	0.620	-	0.380	
**MD of left parietal part**	ACE	4	84.47	-27.53	-	0.250	0	0.750	AE
	CE	3	84.42	-29.23	0.62	-	0.150	0.850	
	AE	3	84.47	-29.53	1.00	0.250	-	0.750	
**MD of left occipital part**	ACE	4	-166.90	-278.90	-	0.367	0	0.633	AE
	CE	3	-165.26	-279.26	0.20	-	0.189	0.811	
	AE	3	-166.90	-280.90	1.00	0.367	-	0.633	

Table S4: Heritability of MD in the right IFOF and its lobar segments.

	Model	ep	-2LL	AIC	p	a^2	c^2	e^2	Best model
**MD of right IFOF**	ACE	4	-261.62	-373.62	-	0.424	0	0.576	AE
	CE	3	-260.82	-374.82	0.18	-	0.375	0.625	
	AE	3	-261.62	-375.62	1.00	0.424	-	0.576	
**MD of right frontal part**	ACE	4	-181.74	-293.74	-	0.479	0	0.521	AE
	CE	3	-179.86	-293.86	0.17	-	0.438	0.562	
	AE	3	-181.74	-295.74	1.00	0.479	-	0.521	
**MD of right insular part**	ACE	4	-226.95	-338.95	-	0	0	1.000	E
	CE	3	-226.95	-340.95	1.00	-	0	1.000	
	AE	3	-226.95	-340.95	1.00	0	-	1.000	
	E	2	-226.95	-342.95	1.00	-	-	1.000	
**MD of right temporal part**	ACE	4	-214.13	-326.13	-	0.365	0	0.635	AE
	CE	3	-213.51	-327.51	0.43	-	0.327	0.673	
	AE	3	-214.13	-328.13	1.00	0.365	-	0.635	
**MD of right parietal part**	ACE	4	53.89	-58.11	-	0.229	0	0.771	AE
	CE	3	54.66	-59.33	0.38	-	0.253	0.747	
	AE	3	53.89	-60.11	1.00	0.229	-	0.771	
**MD of right occipital part**	ACE	4	-185.83	-297.83	-	0	0	1.000	E
	CE	3	-185.83	-299.83	1.00	-	0	1.000	

	AE	3	-185.83	-299.83	1.00	0	-	1.000
	E	2	-185.83	-301.83	1.00	-	-	1.000

Table S5: Heritability of the ratio scores of alerting, orienting, and EC

	Model	ep	-2LL	AIC	p	a^2	c^2	e^2	Best model
**Alerting**	ACE	4	-219.51	-331.51	-	0	0	1.000	E
	CE	3	-219.51	-333.51	1.00	-	0	1.000	
	AE	3	-219.51	-333.51	1.00	0	-	1.000	
	E	2	-219.51	-335.51	1.00	-	-	1.000	
**Orienting**	ACE	4	-185.62	-297.62	-	0.464	0	0.536	AE
	CE	3	-183.82	-297.82	0.18	-	0.315	0.685	
	AE	3	-185.62	-299.62	1.00	0.464	-	0.536	
**EC**	ACE	4	-161.58	-273.58	-	0.375	0	0.625	AE
	CE	3	-160.43	-274.43	0.28	-	0.262	0.738	
	AE	3	-161.58	-275.58	1.00	0.375	-	0.625	
